# Characteristics of diabetic and non-diabetic carpal tunnel syndrome in terms of clinical, electrophysiological, and Sonographic features: a cross-sectional study

**DOI:** 10.1186/s12891-023-06881-1

**Published:** 2023-09-16

**Authors:** Dougho Park, Sang-Eok Lee, Jae Man Cho, Joong Won Yang, ManSu Kim, Heum Dai Kwon

**Affiliations:** 1 Department of Medical Science and Engineering, School of Convergence Science and Technology, Pohang University of Science and Technology, Pohang, Republic of Korea; 2 Department of Rehabilitation Medicine, Pohang Stroke and Spine Hospital, 352, Huimang-daero, Pohang, 37659 Republic of Korea; 3 Department of Neurosurgery, Pohang Stroke and Spine Hospital, Pohang, Republic of Korea

**Keywords:** Carpal tunnel syndrome, Diabetic complications, Electrodiagnosis, Neurologic manifestations, Ultrasonography

## Abstract

**Background:**

Although diabetes is considered a major risk factor for carpal tunnel syndrome (CTS), the characteristics of diabetic CTS have not been fully understood.

**Objective:**

This study is aimed at evaluation of the clinical, electrophysiological, and ultrasonographic findings of non-diabetic and diabetic CTS.

**Methods:**

This retrospective, cross-sectional study included patients diagnosed with CTS. Patient age, sex, involved side, body mass index, clinical and electrophysiological findings, and median nerve cross-sectional area (CSA) were identified. Diabetes was identified through patient or guardian interviews, medical records, and medication history. Linear and binary logistic regression models were established to confirm the associations between the electrophysiological findings, median nerve CSA, and clinical outcomes. Covariates, such as age, sex, body mass index, diabetes, symptom duration, and thenar muscle weakness were adjusted.

**Results:**

Out of the 920 hands, 126 and 794 belonged to the diabetic and non-diabetic CTS groups, respectively. The patients were significantly older in the diabetic CTS group (*P* < 0.001). The rate of thenar weakness in the diabetic CTS group was also significantly higher than that in the non-diabetic CTS group (*P* = 0.009). The diabetic CTS group had a more severe electrodiagnostic grade (*P* = 0.001). The prolonged onset latency of the compound motor nerve action potential (CMAP) and median nerve CSA were well associated with the degree of clinical symptoms. Increased median nerve CSA was significantly associated with prolonged CMAP onset latency (*β* = 0.64; *P* = 0.012), prolonged transcarpal latency (*β* = 0.95; *P* = 0.044), and decreased CMAP amplitude (*β* = -0.17; *P* = 0.002) in the non-diabetic CTS group.

**Conclusion:**

Diabetic CTS had more profound electrophysiological abnormalities. Distal motor latency and median nerve CSA were not only associated with each other, but also with clinical symptoms. Further studies are needed to investigate the pathophysiological mechanisms underlying diabetic CTS.

**Supplementary Information:**

The online version contains supplementary material available at 10.1186/s12891-023-06881-1.

## Introduction

Carpal tunnel syndrome (CTS) is a common entrapment neuropathy [1]. CTS presents various symptoms depending on the severity; with disease progression, the degree of early sensory symptoms, such as paresthesia, numbness, and neuropathic pain increases. In severe CTS, motor symptoms such as thenar muscles weakness and atrophy also appear [2]. Diagnosis using electrodiagnostic tests and severity classification are based on nerve conduction studies along with clinical symptoms [3, 4]. In addition, previous reports have attempted to identify CTS and classify its severity using ultrasonography, which mainly measures the cross-sectional area (CSA), flattening ratio of the median nerve, and palmar bowing of the flexor retinaculum [5]. These two modalities have been used to diagnose CTS and determine the appropriate treatment for each stage [6].

Diabetes is a risk factor and the incidence of CTS is higher in patients with diabetes than in the general population [7, 8]. This is because the susceptibility to nerve compression can increase in patients with diabetes [9]. Mechanisms, such as increased endo-neural pressure, decreased density of myelinated fibers, and stiffness of the transcarpal ligament contribute to increased susceptibility to nerve compression [10, 11]. However, despite several previous studies on CTS and diabetes, results are still inconclusive regarding the differences in clinical, electrophysiological, and ultrasonographic findings between diabetic and non-diabetic CTS [12]. Moreover, the effects of diabetes on median nerve entrapment remain unclear [13].

In this study, we hypothesized that diabetic and non-diabetic CTS had different clinical, electrophysiological, and sonographic features. Therefore, we aimed to elucidate these features in both diabetic and non-diabetic CTS groups. We compared these findings in CTS patients with and without diabetes. Additionally, a stratified subgroup was analyzed based on severity to identify whether the differences between the two groups were valid. Finally, we investigated whether the electrophysiological findings and median nerve CSA were associated with the severity of clinical symptoms in the diabetic and non-diabetic CTS groups.

## Materials and methods

### Study design, setting, sampling, and participants

**Fig. 1:**
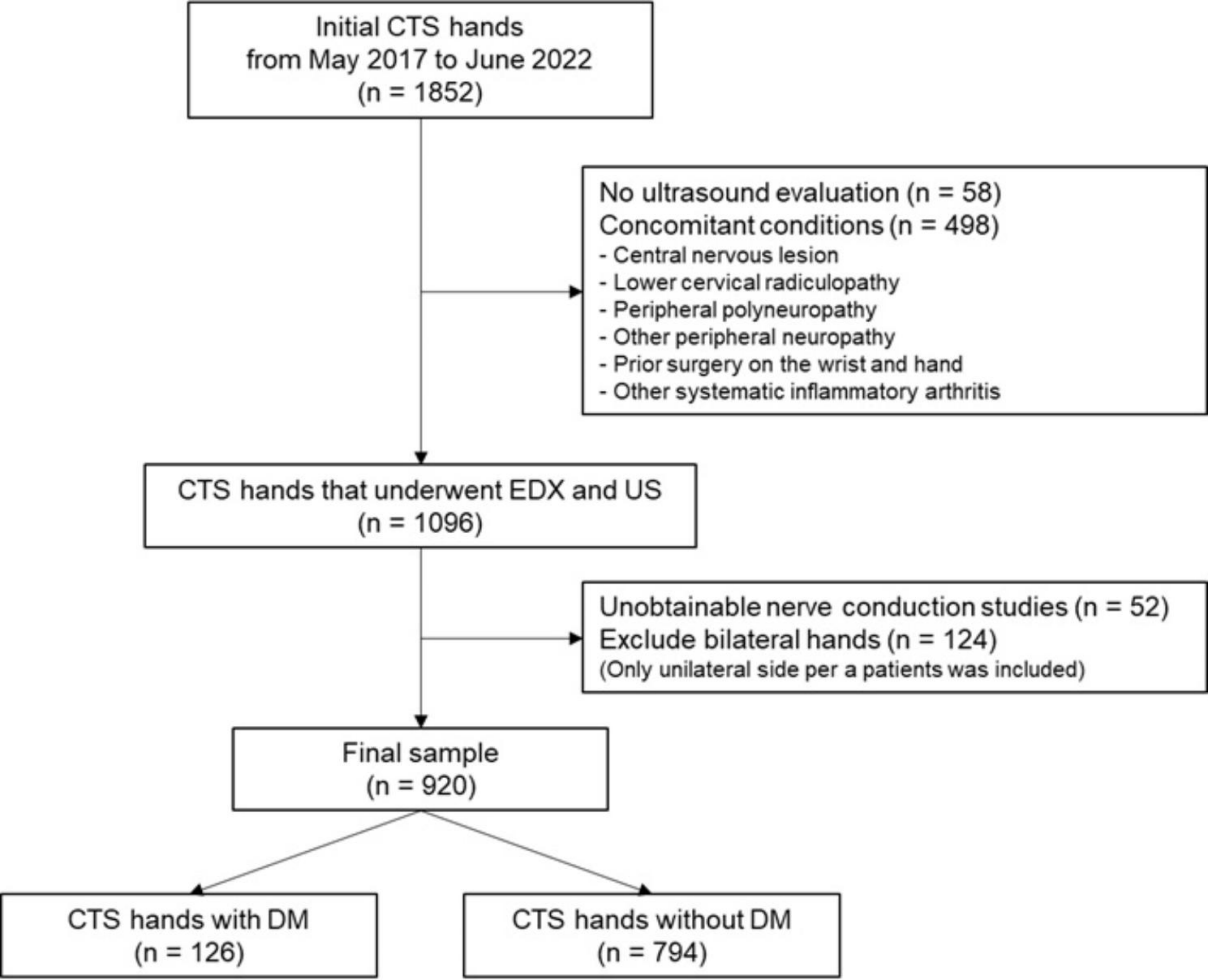
Flowchart of patient inclusion

This was a retrospective cross-sectional study of patients diagnosed with CTS at a single hospital between May 2017 and December 2022. We defined the diagnosis of CTS as follows: (1) showing one or more symptoms or signs corresponding to the CTS diagnosis criteria [14] and (2) any abnormal findings in the sensitivity tests of electrodiagnosis for CTS, including transcarpal latency, lumbrical-interosseous comparison, and ring finger studies [15]. We excluded cases in which median nerve ultrasonography was not performed on the initial samples. Moreover, we excluded the following concurrent conditions: central nervous system lesions, lower cervical radiculopathy, peripheral polyneuropathy, other peripheral neuropathies, prior surgery on the wrist and hand, and other systemic inflammatory arthritis. For rigorous statistical analysis, we included only unilateral hands in the case of bilateral CTS, and block randomization was performed in bilateral CTS cases so that the right and left hands were excluded evenly. We obtained the data of 920 unilateral hands with CTS and divided them into diabetic and non-diabetic CTS groups. G*Power version 3.1.9.7 was used to calculate the sample size. First, we calculated the effect size of the difference between two independent groups when the sample size was 126:794. Type 1 error probability was set to 0.05 and power to 0.8. In addition, we estimated the sample size for a two-tailed correlation with 0.4 effect size, 0.05 type 1 error probability, and 0.95 power [16]. A flowchart of the subject inclusion process is shown in Fig. 1.

The Institutional Review Board of Pohang Stroke and Spine Hospital reviewed and approved this study (approval number: PSSH0475-202303-HR-004-01). Owing to the retrospective study design, the Institutional Review Board approved the omission of informed consent. This study was conducted in compliance with the principles of the Declaration of Helsinki.

### Clinical variables

We identified the patients’ age, sex, affected side, and body mass index at the time of CTS diagnosis. Diabetes was identified through patient or guardian interviews, medical records, and medication history. Cases of diabetic polyneuropathy were excluded. Through patient interviews, we confirmed the timing and duration of symptom onset. The degree of subjective symptoms of the patients was confirmed using a numeric rating scale (NRS) for pain. Provocative night pain and thenar weakness were evaluated.

### Electrodiagnostic and sonographic measurements

Experienced physiatrists performed all electrodiagnostic evaluations using Sierra®wave (Cadwell, Kennewick, WA, USA). All tests were performed with the patients lying down. The examination room temperature was set at 23 − 25 °C.

Based on previous electrodiagnostic classifications [4, 17], we defined mild and moderate-to-severe CTS groups based on the nerve conduction study results for stratified subgroup analysis. The mild group included patients with abnormal results in the sensory nerve conduction studies but normal results in the motor nerve conduction studies. In contrast, the moderate-to-severe group included patients with abnormal findings in the motor nerve conduction studies. The detailed methods for the electrodiagnosis of CTS have been described previously [18, 19].

We identified median nerve CSA as a sonographic parameter. All sonographic evaluations were performed by experienced physiatrists at the time of index electrodiagnosis. The CSA of the median nerve was measured using a transverse view at the pisiform and scaphoid levels (just proximal to the carpal tunnel level) [20]. All tests were conducted using the iU22 equipment (Philips, Bothell, WA, USA) and a linear probe (12–5 Hz).

### Statistical analysis

All statistical analyses were performed using R software version 4.2.2 (R Core Team, The R Foundation for Statistical Computing, Vienna, Austria). Statistical significance was defined as *P*-value < 0.05.

Continuous variables were tested for normality through the Anderson-Darling test and expressed as median (interquartile range). The Wilcoxon rank-sum test was then applied to compare the groups. Categorical variables are expressed as frequency and proportion and the chi-squared test was applied for comparison between groups. Propensity score matching (PSM) was conducted using the “MatchIt” R software package for sensitivity analyses [21]. Age and symptom duration were used for logistic regression, and nearest-neighbor method was used without replacement. The matching ratio was 1:2. Linear and binary logistic regression analyses were used to examine the associations between the electrophysiological findings, median nerve CSA, and clinical outcomes. Covariates, such as age, sex, body mass index, and diabetes were adjusted for in all models. For the model regarding the direct association among the median nerve CSA and electrophysiological findings, we adjusted for age, sex, body mass index, symptom duration, and thenar muscle weakness. We confirmed the multicollinearity of the models based on a variable inflation factor of < 10 and performed a complete case analysis of all models.

## Results

### Baseline characteristics

Of the 920 hands, 126 were in the diabetic CTS group and 794 in the non-diabetic CTS group. The patients in the diabetic CTS group were significantly older (61.5 [56.0–68.0] vs. 57.0 [51.0–64.0] years old; *P* < 0.001). The rate of thenar weakness in the diabetic CTS group was 22.2%, which was significantly higher than 13.0% in the non-diabetic CTS group (*P* = 0.009). The diabetic CTS group showed higher proportion of moderate-to-severe electrodiagnostic grades (*P* = 0.001) with significant differences from the non-diabetic CTS group in all nerve conduction study parameters. However, the CSA of the median nerve was not significantly different between the two groups. After PSM, there was no significant difference between the non-diabetic and diabetic CTS groups regarding age and symptom duration (Table 1).

**Table 1:** Baseline characteristics in all included patients

Variables	CTS with DM (n = 126)		Before PSM			After PSM	
			CTS without DM (n = 794)	P-value		CTS without DM (n = 252)	P-value
Age, years	61.5 (56.0–68.0)		57.0 (51.0–64.0)	< 0.001		61.0 (56.0–68.0)	0.723
Male, n (%)	51 (40.5)		308 (38.8)	0.793		100 (39.7)	0.970
Right hand, n (%)	68 (54.0)		396 (49.9)	0.448		122 (48.4)	0.363
Body mass index, kg/m2	24.9 (22.7–26.7)		24.1 (22.3–26.7)	0.130		24.1 (22.8–26.7)	0.330
Symptom duration, months	5.0 (2.0–12.0)		4.0 (2.0–10.0)	0.125		4.0 (1.0–12.0)	0.144
NRS of pain	4.0 (3.0–6.0)		4.0 (3.0–5.0)	0.480		4.0 (3.0–5.0)	0.620
Night pain, n (%)	60 (47.6)		304 (38.3)	0.058		91 (36.1)	0.410
Thenar weakness, n (%)	28 (22.2)		103 (13.0)	0.009		32 (12.7)	0.025
Electrophysiological findings							
CMAP latency, ms	4.0 (3.7–4.6)		3.8 (3.4–4.3)	< 0.001		3.9 (3.5–4.4)	0.011
CMAP amplitude, mV	7.4 (5.7–8.7)		7.9 (6.5–9.7)	0.002		7.6 (6.1–9.4)	0.082
SNAP latency, ms	3.5 (3.2–3.9)		3.2 (3.0–3.6)	< 0.001		3.3 (3.0–3.6)	< 0.001
SNAP amplitude, uV	15.9 (11.7–21.6)		21.4 (15.5–27.1)	< 0.001		20.4 (15.1–24.6)	< 0.001
Transcarpal latency, ms	2.2 (1.9–2.6)		2.0 (1.8–2.3)	< 0.001		1.9 (1.8–2.3)	< 0.001
Severity grades, n (%)				0.001			0.018
Mild^a^	49 (38.9)		440 (55.4)			132 (52.4)	
Moderate to Severe^b^	77 (61.1)		354 (44.6)			120 (47.6)	
Ultrasonographic finding							
Cross sectional area, mm2	15.0 (12.0–17.0)		14.0 (12.0–17.0)	0.241		14.0 (12.0–17.0)	0.391

^a^sensory abnormalities only

^b^with motor abnormalities

Abbreviations: CMAP, compound motor nerve action potential; DM, diabetes mellitus; NRS, numeric rating scale; PSM, propensity score matching; SNAP, sensory nerve action potential

For the subgroup analysis, we stratified the patients according to electrodiagnostic severity. The mild group contained 49 and 440 hands with diabetic and non-diabetic CTS, respectively. The mild group showed significant differences in sensory nerve action potential (SNAP) parameters between the two groups; the onset latency was significantly longer (*P* = 0.004) and the amplitude was significantly lower (*P* = 0.033) in the diabetic CTS group. Furthermore, the transcarpal latency was prolonged in the diabetic CTS group (*P* = 0.014). There was no significant difference in the onset latency and amplitude of the compound motor nerve action potential (CMAP) between the two groups, and there was no difference in the median CSA between the two groups (Table 2). Meanwhile, 77 and 354 hands with diabetic and non-diabetic CTS were included in the moderate-to-severe group, respectively. There was a significant difference between the two groups in the onset latency of SNAP (*P* = 0.047) but not in CMAP. Further, significantly lower amplitudes of SNAP and CMAP were found in the diabetic CTS group (*P* = 0.027 and *P* = 0.001, respectively). The transcarpal latency and median nerve CSA were not significantly different between the two groups (Table 3).

**Table 2:** Baseline characteristics of carpal tunnel syndrome in the mild group

Variables	CTS with DM (n = 49)	CTS without DM (n = 440)	P-value
Age, years	60.0 (54.0–66.0)	57.0 (51.0–63.0)	0.069
Male, n (%)	19 (38.8)	173 (39.3)	> 0.999
Right hand, n (%)	26 (53.1)	234 (53.2)	> 0.999
Body mass index, kg/m2	24.8 (23.0–26.7)	23.6 (21.8–26.0)	0.011
Symptom duration, months	2.0 (1.0–3.0)	3.0 (1.0–5.0)	0.026
NRS of pain	3.0 (2.0–4.0)	3.0 (2.0–4.0)	0.182
Night pain, n (%)	11 (22.4)	84 (19.1)	0.709
Thenar weakness, n (%)	0 (0.00)	0 (0.00)	NA
Electrophysiological findings			
CMAP latency, ms	3.6 (3.4–3.8)	3.5 (3.3–3.8)	0.400
CMAP amplitude, mV	8.0 (7.0–9.7)	8.6 (7.0–10.1)	0.617
SNAP latency, ms	3.2 (3.0–3.3)	3.0 (2.9–3.2)	0.004
SNAP amplitude, uV	21.5 (15.7–25.9)	23.8 (19.2–29.2)	0.033
Transcarpal latency, ms	1.9 (1.8–2.0)	1.8 (1.7–2.0)	0.014
Ultrasonographic finding			
Cross sectional area, mm2	13.0 (11.0–15.0)	13.0 (11.0–15.0)	0.846

Abbreviations: CMAP, compound motor nerve action potential; DM, diabetes mellitus; NA, not applicable; NRS, numeric rating scale; SNAP, sensory nerve action potential

**Table 3:** Baseline characteristics of carpal tunnel syndrome in the moderate-to-severe group

Variables	CTS with DM (n = 77)	CTS without DM (n = 354)	P-value
Age, years	63.0 (56.0–69.0)	57.0 (51.0–64.0)	< 0.001
Male, n (%)	32 (41.6)	135 (38.1)	0.667
Right hand, n (%)	42 (54.6)	162 (45.8)	0.203
Body mass index, kg/m2	24.9 (22.6–26.6)	24.9 (23.1–27.3)	0.469
Symptom duration, months	9.0 (4.0–23.0)	7.0 (3.0–12.0)	0.098
NRS of pain	5.0 (4.0–6.0)	5.0 (4.0–7.0)	0.419
Night pain, n (%)	49 (63.64)	220 (62.15)	0.909
Thenar weakness, n (%)	28 (36.4)	103 (29.1)	0.263
Electrophysiological findings			
CMAP latency, ms	4.5 (4.2–5.0)	4.4 (4.1–4.8)	0.302
CMAP amplitude, mV	6.9 (4.9–7.8)	7.2 (5.8–8.8)	0.027
SNAP latency, ms	3.8 (3.5–4.1)	3.6 (3.3–4.1)	0.047
SNAP amplitude, uV	13.7 (9.8–17.8)	17.6 (11.4–23.9)	0.001
Transcarpal latency, ms	2.4 (2.2–2.8)	2.3 (2.0–2.8)	0.116
Ultrasonographic finding			
Cross sectional area, mm2	16.0 (14.0–18.0)	16.0 (14.0–18.0)	0.597

Abbreviations: CMAP, compound motor nerve action potential; DM, diabetes mellitus; NRS, numeric rating scale; SNAP, sensory nerve action potential

### Clinical outcomes and their associations in the entire CTS group

The linear and logistic regression models for all CTS cases are presented in Supplementary Material 1: Tables S1 and S2. Symptom duration showed a significant association with prolonged CMAP onset latency (*β* = 1.94; *P* < 0.007) and increased median nerve CSA (*β* = 0.26; *P* = 0.001). NRS of pain was significant association with prolonged CMAP onset latency (*β* = 0.37; *P* < 0.003), transcarpal latency (*β* = 0.49; *P* = 0.018), and increased median nerve CSA (*β* = 0.11; *P* < 0.001). Prolonged CMAP onset latency (adjusted odds ratio [aOR], 1.91; 95% confidence interval [CI], 1.35–2.70; *P* < 0.001) and increased median nerve CSA (aOR, 1.13; 95% CI, 1.08–1.18; *P* < 0.001) were significantly associated with provocative night pain. Further, prolonged CMAP onset latency (aOR, 1.76; 95% CI, 1.18–2.63; *P* = 0.006), prolonged transcarpal latency (aOR, 2.13; 95% CI, 1.04–4.34; *P* = 0.038), and increased median nerve CSA (aOR, 1.21; 95% CI, 1.14–1.29; *P* < 0.001) were significantly associated with the risk of thenar weakness.

## Clinical outcomes and their associations in the non-diabetic CTS group

In the non-diabetic CTS group, symptom duration showed a significant relationship with prolonged CMAP onset latency (*β* = 1.59; *P* = 0.007) and increased median nerve CSA (*β* = 0.27; *P* < 0.001). NRS of pain showed a significant association with prolonged CMAP onset latency (*β* = 0.36; *P* = 0.003) and transcarpal latency (*β* = 0.49; *P* = 0.026). Increased median nerve CSA (*β* = 0.11; *P* < 0.001) was also significantly associated with NRS of pain (Table 4). Similarly, prolonged CMAP onset latency (adjusted aOR, 1.93; 95% CI, 1.31–2.84; *P* < 0.001) and increased median nerve CSA (aOR, 1.13; 95% CI, 1.08–1.31; *P* < 0.001) were found to increase the risk of provocative night pain significantly. Further, prolonged CMAP onset latency (aOR, 1.98; 95% CI, 1.25–3.13; *P* = 0.004) and increased median nerve CSA (aOR, 1.23; 95% CI, 1.15–1.32; *P* < 0.001) were associated with the risk of thenar weakness (Table 5).

**Table 4:** Multivariable linear regression models for symptom duration and subjective pain scale in both diabetic and non-diabetic carpal tunnel syndrome

Groups	Outcomes	Variables	*β* ^a^	SE	*P*-value
Non-diabetic	Symptom duration (months)	CMAP onset latency, ms	1.59	0.58	0.007
		CMAP amplitude, mV	-0.2	0.13	0.132
		SNAP onset latency, ms	2.18	0.92	0.018
		SNAP amplitude, µV	-0.02	0.02	0.270
		Transcarpal latency, ms	0.53	1.08	0.624
		Cross-sectional area, mm^2^	0.27	0.08	< 0.001
		Final model = -10.26 + 3.23CMAP onset latency + 0.31Cross-sectional area			
	NRS of pain	CMAP onset latency, ms	0.36	0.12	0.003
		CMAP amplitude, mV	-0.004	0.03	0.876
		SNAP onset latency, ms	0.25	0.19	0.186
		SNAP amplitude, µV	-0.004	0.004	0.311
		Transcarpal latency, ms	0.49	0.22	0.026
		Cross-sectional area, mm^2^	0.11	0.02	< 0.001
		Final model = -0.54 + 0.44CMAP onset latency + 0.67transcarpal latency + 0.11Cross-sectional area			
Diabetic	Symptom duration (months)	CMAP onset latency, ms	4.63	1.80	0.012
		CMAP amplitude, mV	0.19	0.44	0.670
		SNAP onset latency, ms	-6.45	3.96	0.106
		SNAP amplitude, µV	-0.07	0.12	0.595
		Transcarpal latency, ms	10.33	4.33	0.019
		Cross-sectional area, mm^2^	0.23	0.27	0.394
		Final model = -20.39 + 4.08CMAP onset latency + 5.55transcarpal latency			
	NRS of pain	CMAP onset latency, ms	0.45	0.29	0.129
		CMAP amplitude, mV	-0.06	0.07	0.375
		SNAP onset latency, ms	-0.06	0.64	0.900
		SNAP amplitude, µV	-0.02	0.02	0.252
		Transcarpal latency, ms	0.47	0.70	0.506
		Cross-sectional area, mm^2^	0.13	0.04	0.006
		Final model = 1.31 + 0.20Cross-sectional area			

^a^adjusted with age, sex, and body mass index

Abbreviations: CMAP, compound motor nerve action potential; DM, diabetes mellitus; NRS, numerical rating scale; SE, standard error; SNAP, sensory nerve action potential

**Table 5:** Multivariable logistic regression models for night pain and thenar weakness in both diabetic and non-diabetic carpal tunnel syndrome

Groups	Outcomes	Variables	OR^a^	95% CI	*P*-value
Non-diabetic	Night pain	CMAP onset latency, ms	1.93	1.31–2.84	< 0.001
		CMAP amplitude, mV	0.97	0.90–1.05	0.446
		SNAP onset latency, ms	0.75	0.42–1.36	0.343
		SNAP amplitude, µV	0.99	0.97–1.01	0.410
		Transcarpal latency, ms	1.91	0.93–3.94	0.078
		Cross-sectional area, mm^2^	1.13	1.08–1.31	< 0.001
	Thenar weakness	CMAP onset latency, ms	1.98	1.25–3.13	0.004
		CMAP amplitude, mV	0.99	0.88–1.11	0.884
		SNAP onset latency, ms	1.04	0.53–2.04	0.908
		SNAP amplitude, µV	1.00	0.99–1.02	0.620
		Transcarpal latency, ms	1.79	0.85–3.76	0.124
		Cross-sectional area, mm^2^	1.23	1.15–1.32	< 0.001
Diabetic	Night pain	CMAP onset latency, ms	1.88	0.80–4.43	0.147
		CMAP amplitude, mV	0.99	0.82–1.12	0.956
		SNAP onset latency, ms	1.47	0.22–9.84	0.694
		SNAP amplitude, µV	0.98	0.92–1.04	0.428
		Transcarpal latency, ms	1.19	0.16–9.16	0.866
		Cross-sectional area, mm^2^	1.13	0.99–1.29	0.063
	Thenar weakness	CMAP onset latency, ms	1.27	1.18–2.63	0.602
		CMAP amplitude, mV	1.03	0.89–1.09	0.792
		SNAP onset latency, ms	0.55	0.56–2.01	0.578
		SNAP amplitude, µV	0.93	0.98–1.02	0.167
		Transcarpal latency, ms	6.71	0.60–75.34	0.123
		Cross-sectional area, mm^2^	1.13	0.97–1.32	0.108

^a^adjusted with age, sex, and body mass index

Abbreviations: CI, confidence interval; CMAP, compound motor nerve action potential; DM, diabetes mellitus; OR, odds ratio; SNAP, sensory nerve action potential

In the matched non-diabetic CTS group, symptom duration showed a significant association with prolonged CMAP onset latency (*β* = 2.51; *P* = 0.035), while NRS of pain showed a significant association with both prolonged CMAP onset latency (*β* = 0.52; *P* = 0.015) and median nerve CSA (*β* = 0.06; *P* = 0.025) (Supplementary Material 1: Table S3). Meanwhile, increased median nerve CSA (aOR, 1.14; 95% CI, 1.05–1.25; *P* = 0.002) significantly increased the risk of provocative night pain. Prolonged CMAP onset latency (aOR, 5.56; 95% CI, 2.09–15.24; *P <* 0.004) and increased median nerve CSA (aOR, 1.16; 95% CI, 1.02–1.33; *P* = 0.028) were also associated with the risk of thenar weakness (Supplementary Material 1: Table S4).

### Clinical outcomes and their associations in the diabetic CTS group

In the diabetic CTS group, symptom duration was strongly associated with prolonged CMAP onset latency (*β* = 4.63; *P* = 0.012) and transcarpal latency (*β* = 10.33; *P* = 0.019). Meanwhile, NRS of pain significantly correlated with the increase in median CSA (*β* = 0.13; *P* = 0.006) (Table 4). Provocative night pain and thenar weakness did not significantly correlate with any electrodiagnostic or sonographic parameters in the diabetic CTS group (Table 5).

### Associations between electrophysiological findings and median nerve CSA

In the non-diabetic CTS group, increased median nerve CSA was significantly associated with prolonged CMAP onset latency (*β* = 0.64; *P* = 0.012) and transcarpal latency (*β* = 0.95; *P* = 0.044). Further, decreased CMAP amplitude (*β* = -0.17; *P* = 0.002) was also significantly associated with increased median nerve CSA (Table 6). In the matched non-diabetic CTS group, increased median nerve CSA was significantly associated with prolonged transcarpal latency (*β* = 1.56; *P* = 0.032) (Supplementary Material 1: Table S5).

**Table 6:** Associations between electrodiagnostic findings and median nerve cross-sectional area

Groups	Outcome	Variables	*β* ^a^	SE	*P*-value
Non-diabetic	Median nerve CSA	CMAP onset latency, ms	0.64	0.26	0.012
		CMAP amplitude, mV	-0.17	0.06	0.002
		SNAP onset latency, ms	0.002	0.40	0.996
		SNAP amplitude, uV	-0.01	0.008	0.055
		Transcarpal latency, ms	0.95	0.47	0.044
Diabetic	Median nerve CSA	CMAP onset latency, ms	0.89	0.62	0.156
		CMAP amplitude, mV	-0.11	0.15	0.455
		SNAP onset latency, ms	0.16	1.35	0.907
		SNAP amplitude, uV	-0.02	0.04	0.613
		Transcarpal latency, ms	0.89	1.49	0.555

^a^adjusted for age, sex, body mass index, symptom duration, and thenar weakness

CMAP, compound motor nerve action potential; CSA, cross-sectional area; SE, standard error; SNAP, sensory nerve action potential

However, no significant association was observed between the electrophysiological findings and median nerve CSA in the diabetic CTS group (Table 6).

## Discussion

This study analyzed the differences in the clinical, electrodiagnostic, and sonographic findings between patients with and without diabetic CTS. We investigated whether the electrophysiological findings and CSA of the median nerve were associated with symptom severity in each group.

Our results confirmed that the diabetic group had a more advanced CTS. Moreover, stratified analyses revealed that SNAP abnormalities were more prominent in diabetic CTS in the mild group. In contrast, both CMAP and SNAP amplitudes, rather than latency, were identified as characteristics that differentiated diabetic CTS from non-diabetic CTS in the moderate-to-severe group. We inferred that this reflected the progression of neuropathy. In the early stages of neuropathy, sensory nerves are more vulnerable, with dominating demyelinating features. As the disease progresses, motor neurons get involved, and axonopathic features appear [22–24]. In this study, the significant differences in the electrophysiological findings between the diabetic and non-diabetic groups could be attributed to the fact that vulnerability to neural compression is greater in the case of diabetic CTS [25, 26].

Previous studies have found inconsistent results regarding whether the comorbidity of diabetes in CTS causes significant differences in electrophysiological findings. Tony et al. [27] analyzed 36 non-diabetic and 25 diabetic CTS cases and found a significant difference between the two groups in median CMAP amplitude and SNAP latency, but no difference in distal motor latency. In contrast, Kim et al. [28] compared 22 diabetic CTS and 83 non-diabetic CTS cases; however, both CMAP and SNAP showed no significant differences between the two groups. These negative results suggest that unmyelinated thin fibers are more vulnerable to microvascular alterations caused by diabetes [29], whereas electrophysiological examination is a diagnostic tool that mainly evaluates thick myelinated fibers [30]. However, these two previous studies did not conduct additional analyses that considered the severity of CTS. In contrast, our study confirmed that electrophysiological abnormalities were more prominent in diabetic CTS in both mild and moderate-to-severe groups, using a larger sample than in the previous related studies. Therefore, our results are reliable and valid. Kudo et al. [31] reported that distal motor latency was the most reliable factor reflecting clinical symptoms in a study of 61 patients with unilateral CTS. Our study supports their findings; CMAP onset latency was generally correlated with all clinical outcomes in non-diabetic CTS and was related to symptom duration in diabetic CTS.

Broadly, median CSA is associated with CTS severity [32]. However, the effect of diabetes on the CSA of the median nerve in CTS has shown inconsistent results previously. Kotb et al. [33] reported a significant difference in the median nerve CSA between 44 diabetic and 46 non-diabetic CTS cases. However, other studies reported no significant difference in median nerve CSA between diabetic and non-diabetic CTS groups [28, 34], which is consistent with our findings. Furthermore, whether an enlarged median nerve is useful for grading the CTS severity remains controversial [35, 36]. In our results, median nerve CSA was generally associated with clinical outcomes, and this tendency was more prominent in the non-diabetic group. In contrast, in the diabetic CTS group, only the NRS score for pain showed a significant association with the CSA. Combining our results with those of previous studies, we suggest that it is difficult to identify symptoms and causality in related to median nerve enlargement in diabetic CTS compared with non-diabetic CTS [37].

Additionally, our results suggested that median nerve CSA and electrophysiological findings showed a significant association in the non-diabetic group, but not in the diabetic group. In previous studies analyzing the diagnostic accuracy of CTS in patients with diabetic neuropathy, it was generally reported that abnormal results of nerve conduction studies and CSA enlargement were associated [38, 39]. The difference in these results may be because of the variations in the target group, since we excluded diabetic polyneuropathy from the target group in advance. In addition, some previous studies reported that sonographic findings were not related to electrodiagnostic CTS severity grading [40, 41]. The results of our diabetic CTS group supported these contradictory results to some extent. The fact that the median nerve enlargement can be less prominent in the chronic and severe phases was the main reason for the results showing that the median nerve CSA and electrophysiological findings were not related in the diabetic CTS group, which showed advanced severity in our cohort. Furthermore, distal motor latency and median nerve CSA were correlated with each other and with clinical symptoms.

This study has several strengths compared with previous studies. First, it analyzed a relatively large sample size compared with previous related studies. Moreover, we utilized data based on the clinical pathway in which clinical, electrodiagnostic, and ultrasound examinations were performed almost simultaneously when a patient visited the hospital. Consequently, we could analyze various clinical outcomes as dependent variables. Therefore, the characteristics of diabetic and non-diabetic CTS can comprehensively be described as multifaceted. We further validated our findings by performing sensitivity analyses by matching some of the non-diabetic CTS. Finally, considering the broad spectrum of CTS itself, our study was significant in that we presented focused results that were adjusted for the severity and presence of diabetes through stratified subgroup analyses.

The limitations of this study were as follows. First, this study was a single hospital-based retrospective study. Since we excluded cases of diabetic polyneuropathy and unobtainable nerve conduction study results, it is difficult to state whether the overall characteristics of diabetic CTS were appropriately reflected in this study. Glycemic control is a factor that explains the degree of diabetic neuropathy [42]; however, we did not report the degree of glycemic control in patients with diabetes. There was an imbalance in the sample size of diabetic and non-diabetic CTS hands in our cohort, which may have lowered the statistical power. Because we conducted the study with a cohort in which CTS had already been confirmed clinically and electrodiagnostically, it was difficult to evaluate the effect of diabetes on the initial diagnosis of CTS and the validity of each diagnostic method for diabetic CTS. Furthermore, since this study utilized patient or guardian interviews, medical records, and medication history to confirm the presence or absence of diabetes, and specific information on diabetes is limited. Finally, examiner bias may exist since the examiners for the median nerve CSA measurements and electrodiagnostic evaluations were not blinded.

## Conclusion

This study analyzed the differences in the clinical, electrophysiological, and sonographic findings between patients with and without diabetic CTS. In addition, we identified associations between each result and the severity of the clinical symptoms. Diabetic CTS has more profound electrophysiological abnormalities, showing sensory-prominent demyelinating features in the early stages of the disease that progress to sensory and motor axonopathy features as the disease progresses. In addition, distal motor latency and median nerve CSA not only correlated with each other but also correlated well with clinical symptoms. The relationship between clinical, electrophysiological, and sonographic findings was more prominent in the non-diabetic CTS group than in the diabetic CTS group. Future studies to determine the cause of this phenomenon and better understanding the underlying pathophysiological mechanism of diabetic CTS are warranted.

### Electronic supplementary material

Below is the link to the electronic supplementary material.

Supplementary Material 1

Supplementary Material 2

## Data Availability

The dataset supporting the conclusions of this article is provided in additional files.

## Abbreviations

aOR: Adjusted odds ratio
CI: Confidence interval
CMAP: Compound motor nerve action potential
CSA: Cross-sectional area
CTS: Carpal tunnel syndrome
NRS: Numeric rating scale
PSM: Propensity score matching
SNAP: Sensory nerve action potential
