# Supplementary material for: Characteristics of diabetic and non-diabetic carpal tunnel syndrome in terms of clinical, electrophysiological, and Sonographic features: a cross-sectional study
Source: BMC Musculoskelet Disord. 2023 Sep 16;24:739. doi: 10.1186/s12891-023-06881-1 (PMC10504773; doi:10.1186/s12891-023-06881-1)
Supplement: Supplementary file 1 — Supplementary Material 1 [file 12891_2023_6881_MOESM1_ESM.docx]

**Supplementary Tables**

**Table S1.** Multivariable linear regression models for symptom duration and subjective pain scale scores in all patients.

**Table S2.** Multivariable logistic regression models for night pain and thenar weakness in all patients.

**Table S3.** Multivariable linear regression models for symptom duration and subjective pain scale in the matched non-diabetic carpal tunnel syndrome group.

**Table S4.** Multivariable logistic regression models for night pain and thenar weakness in the matched non-diabetic carpal tunnel syndrome group.

**Table S5.** Associations between electrodiagnostic findings and median nerve cross-sectional area in the matched non-diabetic carpal tunnel syndrome group.

**Table S1.** Multivariable linear regression models for symptom duration and subjective pain scale in all patients. Symptom duration showed a significant association with prolonged CMAP onset latency and increased median nerve CSA. NRS of pain was significant association with prolonged CMAP onset latency, transcarpal latency, and increased median nerve CSA.

| Outcomes | Variables | *β*^a^ | SE | *P*-value |
| --- | --- | --- | --- | --- |
| Symptom duration  (months) | CMAP onset latency, ms | 1.94 | 0.57 | <0.001 |
|  | CMAP amplitude, mV | -0.16 | 0.13 | 0.213 |
|  | SNAP onset latency, ms | 1.51 | 0.92 | 0.103 |
|  | SNAP amplitude, μV | -0.02 | 0.02 | 0.317 |
|  | Transcarpal latency, ms | 1.46 | 1.07 | 0.175 |
|  | Cross-sectional area, mm^2^ | 0.26 | 0.78 | 0.001 |
|  | Final model = -11.84 + 3.71CMAP onset latency + 0.30Cross-sectional area | | | |
| NRS of pain | CMAP onset latency, ms | 0.37 | 0.11 | <0.001 |
|  | CMAP amplitude, mV | -0.01 | 0.02 | 0.632 |
|  | SNAP onset latency, ms | 0.21 | 0.18 | 0.248 |
|  | SNAP amplitude, μV | -0.004 | 0.004 | 0.213 |
|  | Transcarpal latency, ms | 0.49 | 0.21 | 0.018 |
|  | Cross-sectional area, mm^2^ | 0.11 | 0.02 | <0.001 |
|  | Final model = -0.59 + 0.45CMAP onset latency + 0.66transcarpal latency + 0.11Cross-sectional area | | | |

^a^adjusted for age, sex, body mass index, and diabetes mellitus.

Abbreviations: CMAP, compound motor nerve action potential; NRS, numerical rating scale; SE, standard error; SNAP, sensory nerve action potential.

**Table S2.** Multivariable logistic regression models for night pain and thenar weakness in all patients. Prolonged CMAP onset latency and increased median nerve CSA were significantly associated with provocative night pain. Further, prolonged CMAP onset latency, prolonged transcarpal latency, and increased median nerve CSA were significantly associated with the risk of thenar weakness.

| Outcomes | Variables | OR^a^ | 95% CI | *P*-value |
| --- | --- | --- | --- | --- |
| Night pain | CMAP onset latency, ms | 1.91 | 1.35–2.70 | <0.001 |
|  | CMAP amplitude, mV | 0.98 | 0.91–1.05 | 0.497 |
|  | SNAP onset latency, ms | 0.81 | 0.46–1.41 | 0.452 |
|  | SNAP amplitude, μV | 0.99 | 0.97–1.01 | 0.342 |
|  | Transcarpal latency, ms | 1.85 | 0.95–3.61 | 0.071 |
|  | Cross-sectional area, mm^2^ | 1.13 | 1.08–1.18 | <0.001 |
| Thenar weakness | CMAP onset latency, ms | 1.76 | 1.18–2.63 | 0.006 |
|  | CMAP amplitude, mV | 0.98 | 0.89–1.09 | 0.758 |
|  | SNAP onset latency, ms | 1.06 | 0.56–2.01 | 0.847 |
|  | SNAP amplitude, μV | 1.00 | 0.98–1.02 | 0.848 |
|  | Transcarpal latency, ms | 2.13 | 1.04–4.34 | 0.038 |
|  | Cross-sectional area, mm^2^ | 1.21 | 1.14–1.29 | <0.001 |

^a^adjusted for age, sex, body mass index, and diabetes mellitus.

Abbreviations: CI, confidence interval; CMAP, compound motor nerve action potential; OR, odds ratio; SNAP, sensory nerve action potential.

**Table S3.** Multivariable linear regression models for symptom duration and subjective pain scale in the matched non-diabetic carpal tunnel syndrome group. In the matched non-diabetic CTS group, symptom duration showed a significant association with prolonged CMAP onset latency, while NRS of pain showed a significant association with both prolonged CMAP onset latency and median nerve CSA.

| Groups | Outcomes | Variables | *β*^a^ | SE | *P*-value |
| --- | --- | --- | --- | --- | --- |
| Matched Non-diabetic | Symptom duration  (months) | CMAP onset latency, ms | 2.51 | 1.18 | 0.035 |
|  |  | CMAP amplitude, mV | -0.22 | 0.26 | 0.389 |
|  |  | SNAP onset latency, ms | 3.22 | 1.72 | 0.063 |
|  |  | SNAP amplitude, μV | -0.07 | 0.07 | 0.327 |
|  |  | Transcarpal latency, ms | -0.55 | 1.77 | 0.755 |
|  |  | Cross-sectional area, mm^2^ | 0.29 | 0.16 | 0.064 |
|  |  | Final model = -7.72 + 2.51CMAP onset latency | | | |
|  | NRS of pain | CMAP onset latency, ms | 0.52 | 0.21 | 0.015 |
|  |  | CMAP amplitude, mV | 0.01 | 0.05 | 0.810 |
|  |  | SNAP onset latency, ms | 0.33 | 0.31 | 0.284 |
|  |  | SNAP amplitude, μV | -0.003 | 0.01 | 0.833 |
|  |  | Transcarpal latency, ms | 0.52 | 0.32 | 0.105 |
|  |  | Cross-sectional area, mm^2^ | 0.06 | 0.03 | 0.025 |
|  |  | Final model = -1.32 + 0.52CMAP onset latency + 0.06Cross-sectional area | | | |

^a^adjusted with age, sex, and body mass index.

Abbreviations: CMAP, compound motor nerve action potential; DM, diabetes mellitus; NRS, numerical rating scale; SE, standard error; SNAP, sensory nerve action potential.

**Table S4.** Multivariable logistic regression models for night pain and thenar weakness in the matched non-diabetic carpal tunnel syndrome group. Meanwhile, increased median nerve CSA significantly increased the risk of provocative night pain. Prolonged CMAP onset latency and increased median nerve CSA were also associated with the risk of thenar weakness.

| Groups | Outcomes | Variables | OR^a^ | 95% CI | *P*-value |
| --- | --- | --- | --- | --- | --- |
| Matched Non-diabetic | Night pain | CMAP onset latency, ms | 1.55 | 0.79–3.04 | 0.206 |
|  |  | CMAP amplitude, mV | 0.93 | 0.92–1.14 | 0.638 |
|  |  | SNAP onset latency, ms | 1.14 | 0.46–2.86 | 0.779 |
|  |  | SNAP amplitude, μV | 1.03 | 0.99–1.01 | 0.151 |
|  |  | Transcarpal latency, ms | 2.22 | 0.87–5.67 | 0.096 |
|  |  | Cross-sectional area, mm^2^ | 1.14 | 1.05–1.25 | 0.002 |
|  | Thenar weakness | CMAP onset latency, ms | 5.56 | 2.09–15.42 | <0.001 |
|  |  | CMAP amplitude, mV | 1.17 | 0.93–1.47 | 0.193 |
|  |  | SNAP onset latency, ms | 0.89 | 0.28–2.88 | 0.847 |
|  |  | SNAP amplitude, μV | 1.03 | 0.95–1.11 | 0.487 |
|  |  | Transcarpal latency, ms | 1.79 | 0.50–6.42 | 0.372 |
|  |  | Cross-sectional area, mm^2^ | 1.16 | 1.02–1.33 | 0.028 |

^a^adjusted with age, sex, and body mass index.

Abbreviations: CI, confidence interval; CMAP, compound motor nerve action potential; DM, diabetes mellitus; OR, odds ratio; SNAP, sensory nerve action potential.

**Table S5.** Associations between electrodiagnostic findings and median nerve cross-sectional area in the matched non-diabetic carpal tunnel syndrome group. In the matched non-diabetic CTS group, increased median nerve CSA was significantly associated with prolonged transcarpal latency.

| Groups | Outcome | Variables | *β*^a^ | SE | *P*-value |
| --- | --- | --- | --- | --- | --- |
| Matched  Non-diabetic | Median nerve CSA | CMAP onset latency, ms | 0.78 | 0.50 | 0.118 |
|  |  | CMAP amplitude, mV | -0.16 | 0.11 | 0.140 |
|  |  | SNAP onset latency, ms | -0.84 | 0.71 | 0.237 |
|  |  | SNAP amplitude, uV | 0.003 | 0.03 | 0.910 |
|  |  | Transcarpal latency, ms | 1.56 | 0.72 | 0.032 |

^a^adjusted for age, sex, body mass index, symptom duration, and thenar weakness.

Abbreviations: CMAP, compound motor nerve action potential; CSA, cross-sectional area; SE, standard error; SNAP, sensory nerve action potential.
